# Potential of IMU-Based Systems in Measuring Single Rapid Movement Variables in Females with Different Training Backgrounds and Specialization

**DOI:** 10.1155/2020/7919514

**Published:** 2020-06-30

**Authors:** Stefan Marković, Milivoj Dopsaj, Sašo Tomažič, Anton Umek

**Affiliations:** ^1^Faculty of Sport and Physical Education, University of Belgrade, Belgrade 11000, Serbia; ^2^Faculty of Electrical Engineering, University of Ljubljana, Ljubljana 1000, Slovenia; ^3^Institute of Sport, Tourism and Service, South Ural State University, Chelyabinsk 454080, Russia

## Abstract

The aim of this paper is to determine the discriminative potential of the IMU-based system for the measurement of rapid hand movement properties, i.e., relevant kinematic variables in relation to different groups of participants. The measurement of the kinematics of the rapid hand movement was performed using a standard hand tapping test. The sample in this research included a total of 70 female participants and was divided into 3 subsamples. The discriminant analysis has identified two functions, DF_1_ and DF_2_, that explain 91.1 and 8.1% of the variance, respectively. The differences between the examined subsamples originate from the variables grouped in DF_1_, which were statistically significant (*p* ≤ 0.000). In relation to this function, the national volleyball team centroid position was shifted with -1.108 and -1.968 standard deviation values from the control group and youth volleyball team, respectively. The difference between control and Voll_Youth groups was -0.860 standard deviation value. The factors with the greatest discriminative potential among the groups represent the temporal characteristics of the rapid hand movement, i.e., the time elapsed between the onset of the movement and the first and second tap, as defined by the variables *t*_1_ and *t*_2_, respectively. The established findings clearly indicate that IMU sensors are practically applicable in relation to the sensitive measurement of rapid arm movement capability of female athletes.

## 1. Introduction

In recent years, there has been a rapid development of microelectromechanical sensor systems (MEMS). Along with it came the implementation and application of such systems in different professional environments as well as in everyday use [1]. In this context, the system of sport is not an exception, and various wearable sensors have been developed and used in testing, training, and competition in order to provide new, or more in-depth, information regarding different aspects of sports performance. In essence, this reflects more broad tendencies regarding the implementation of new technologies for the purposes of obtaining more sensitive and sport-specific information in relation to the level of achieved preparedness in elite athletes [2].

Miniature inertial measurement unit (IMU) is a typical example of the MEMS technology which has been increasingly used as a means for motion analysis [3] for the purposes of sports science and praxis. Typically, an IMU that incorporates a triaxial accelerometer, gyroscope, and magnetometer is built into a miniature wearable device [4]. This allows measurement of acceleration, angular velocity, and orientation and also permits sensor fusion for tracking of three-dimensional movements to a variable extent of precision. In addition, it is possible to use an IMU in order to obtain relevant information about the temporal characteristics of the analyzed movements [5]. In this case, the sampling frequency of the system determines the level of measurement precision. Primary applications of IMU-based systems in sports training, testing, and competition are related to either concurrent or terminal biomechanical biofeedback [1] or to the assessment of the physical characteristics relevant for performance and injury prevention [6–8].

The development of sports science increasingly requires a multistructured, integrative approach to information gathering in both laboratory and field testing conditions. This requires the application of multiple measurement methods and technologies [9] in order to obtain relevant information regarding the level of achieved physical fitness during different phases of athletes' preparation. In addition to being a basis for assessment, these results can be used for the purposes of calculating the potential of physical abilities and the efficiency of athletes' performance [10, 11]. In this sense, sports science and praxis employ both basic, i.e., universal, and specific testing batteries [12] for permanent and periodical monitoring of physical properties, expressed in nonspecific conditions as well as in specific conditions of competitive stress [13]. Although from the aspect of movement, the system of sport is very complex and diversified, and it can be argued that rapid simple movements are the main form of movements in basically all sports [14]. Accordingly, regardless of the specificity of the testing conditions, it is necessary to provide relevant information about the athletes' potential in this aspect. In this context, volleyball is a typical example of a sport that sets high and complex technical, tactical, and physical requirements for the players. This, in turn, requires overall development of motor abilities and specific motor skills [15] which can be considered a multidimensional, multistage task that requires constant monitoring.

As previously mentioned, IMU-based measurement systems have been increasingly used in different sport settings for various purposes including performance and technique evaluation [16], although their application in measurement of fast hand and arm movements has been fairly limited. In this context, baseball pitching has been the most frequently researched topic due to the high incidence of injuries related to this particular type of throwing motion and the need to accurately measure the dynamics of the involved segments during peak activity in order to quantify relevant aspects of performance [17]. As throwing a baseball and hitting a volleyball are similar in overhead functional demand, although they generate different kinematic patterns [18], IMU-based systems are also applicable in this context and were used in recent studies for classification of volleyball players based on spiking performance and evaluation of wrist speed and as a part of measurement systems used for movement classification [19–21].

In volleyball, high arm speed is a general prerequisite of successful performance, as it is generally required for efficient spiking [22]. Therefore, relevant information regarding the differences between groups in relation to the kinematic characteristics of rapid arm and hand movement can contribute to the better understanding of the stages of athletes' development and potential effects of training and selection process on their capabilities in this regard. Comparison of volleyball players of different age categories but similar competitive ranking within each category and physically active controls (with no volleyball background) can provide insight into some of the attributes that are unique to the players [23] or can serve as a basis for identification of the individuals that are potentially more capable in this regard. In relation to the aforementioned, the hand tapping test was chosen for the purposes of this research as it is not sport-specific and it is widely used as a part of basic test batteries in different sports as well as in testing of basic motor abilities in a nonathlete population.

The aim of this paper is to determine the discriminative potential of the IMU-based system for measurement of rapid hand movement properties, i.e., to define relevant kinematic variables in relation to different groups of participants.

## 2. Materials and Methods

### 2.1. The Research Sample

The sample in this research included a total of 70 female participants. The overall sample was divided into 3 groups, of which one included physically active controls (age = 22.3 ± 1.9 years, BH = 168.8 ± 5.3 cm, BW = 64.5 ± 2.8 kg), while the other two consisted of the members of the Republic of Serbia national volleyball team (age = 24.5 ± 3.5 years, BH = 186.7 ± 4.2 cm, BW = 75.6 ± 2.6 kg) and national-level young volleyball players (age = 16.8 ± 1.8 years, BH = 180.4 ± 6.5 cm, BW = 71.1 ± 3.2 kg), respectively.

### 2.2. Measurement Methods

**Figure 1:**
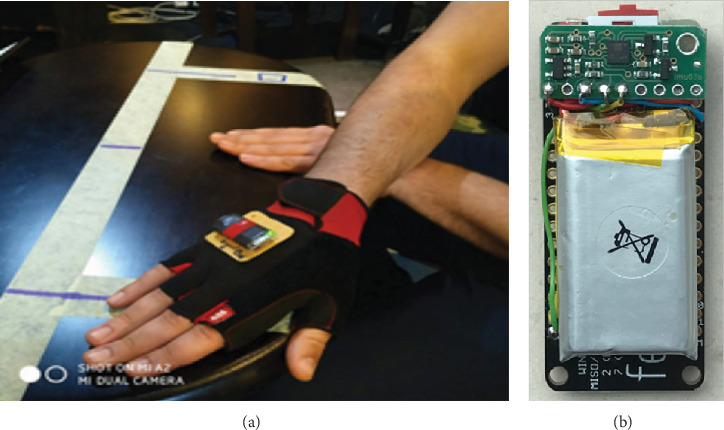
(a) The initial position of the subject's hand with the IMU sensor attached to the glove. (b) A custom-made wireless sensor device (uncovered).

The measurement of the kinematics of the rapid hand movement was performed using a test that represents the gold standard in the measurement of rapid movements of the extremities—standard hand tapping test [9, 24, 25]. This standard test included lateral alternating hand movement between two markers positioned at the 50 cm distance on the table in front of the participant. The test was performed in an upright sitting position with the dominant hand, which was initially placed on the mark at the opposite side, while the nondominant hand was placed at the mark positioned at the midlength of the movement distance, as shown in Figure 1(a). When ready, the subject performed a maximally fast movement. After performing 2 pretest familiarization trials, each participant performed three trials separated with at least 3 minutes of rest [11]. The best result was taken for further statistical processing [26].

For the purposes of this research, we developed a portable measurement system, which allows for quick setup. The wireless sensor device is connected to a laptop running the LabView application. A custom-made wireless sensor device includes an IMU MEMS sensor, a microcontroller with a built-in Wi-Fi communication module, and a LiPo battery for multihour operation.  Figure 1(b) shows a custom-made sensor device without a protective housing. The sensor device is attached to the glove as shown in Figure 1(a). The acceleration in the *Y*-axis corresponds to the line of hand movement, i.e., the line connecting the markers.

The sensor device is equipped with a combined 3D accelerometer and 3D gyroscope (LSM6DS33, STMicroelectronics, Genève, Switzerland) [27]; however, for the purpose of our research, we used only accelerometer data. The sampling frequency is 200 Hz, and the dynamic range of the accelerometer is ±16 g_0_. The wireless sensor device continuously sends data via a Wi-Fi interface while a LabVIEW application is used for acceleration signal processing and kinematic variable data acquisition.

A custom LabView (LabView 2019, National Instruments, Austin, Texas) application was used in order to process the acceleration signal. The LabView application contains a module for receiving accelerometer samples in UDP packets, and the obtained accelerometer signal was filtered with a low-pass Butterworth filter (order = 5, fcof = 40 Hz). The relevant variables in the movement kinematics were automatically identified after the onset of the motion, which was detected when the absolute acceleration exceeded 1.15 g_0_. The application implements automatic threshold and peak detection using predefined SubVIs provided by National Instruments for both *A*_*Y*_ and abs (*A*), thus providing the location and/or magnitude of relevant kinematic and temporal variables. Detection of the acceleration gradient variables was performed using the peak detector SubVI on the signal obtained by derivation of the acceleration over time.

### 2.3. Variables

The following variables acquired from the processed hand acceleration signal were used in order to define the relevant temporal and kinematic characteristics of the movement:

- *t*_1_ is the time from the start of the movement to the first tap of the hand (expressed in s)
- *t*_2_ is the time from the first tap to the second tap of the hand (expressed in s)
- *A*_1_ is the maximal acceleration (expressed as a multiplier of g_0_)
- *A*_2_ is the maximal deceleration (expressed as a multiplier of g_0_)
- GA_1_ is the maximal acceleration gradient (expressed in g_0_·s^−1^)
- GA_2_ is the maximal deceleration gradient (expressed in g_0_·s^−1^)

**Figure 2:**
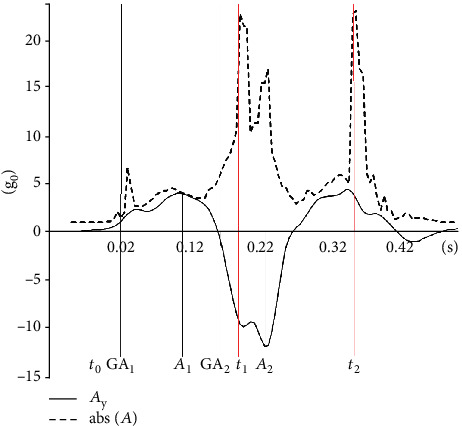
Absolute acceleration (abs) and acceleration in the *Y*- (dominant) axis with the time frame of relevant events.

It should be noted that all acceleration-related variables were measured in the first part of tapping, prior to the first hand tap. The examined variables and the time frame of events are shown on a typical example of the acceleration signal (Figure 2).

### 2.4. Statistical Analysis

For the purposes of this paper, all variables were processed using descriptive statistical analysis in order to determine relevant measures of central tendency, data dispersion, and range (mean, StDev, SEM, cV%, Min and Max) for the respective subsamples. The normality of the distribution of the results was determined by the application of the nonparametric Kolmogorov-Smirnov goodness-of-fit test (K-S Z). The position of centroid groups' location, as a group standardized multivariate score, and the structure of the extracted functions and group differences were defined by discriminant analysis. The level of statistical significance was defined based on the criterion *p* ≤ 0.05 [28]. All data analyses were conducted using Excel 2013 and IBM SPSS v23 statistical software.

## 3. Results and Discussion

Table 1 shows the results of the descriptive statistical analysis of the relevant kinematic variables in relation to the examined groups, as well as the results of the one-sample nonparametric Kolmogorov-Smirnov goodness-of-fit test.

Table 2 shows the summary of the canonical discriminant functions and the results of the general statistical differences between groups in relation to the examined variables.

Table 3 shows the structure matrix of the extracted functions explaining the determined general differences between groups.

Table 4 shows the classification of the group membership in relation to the results of the discriminant analysis based on the relevant kinematic variables of rapid hand movement.

**Figure 3:**
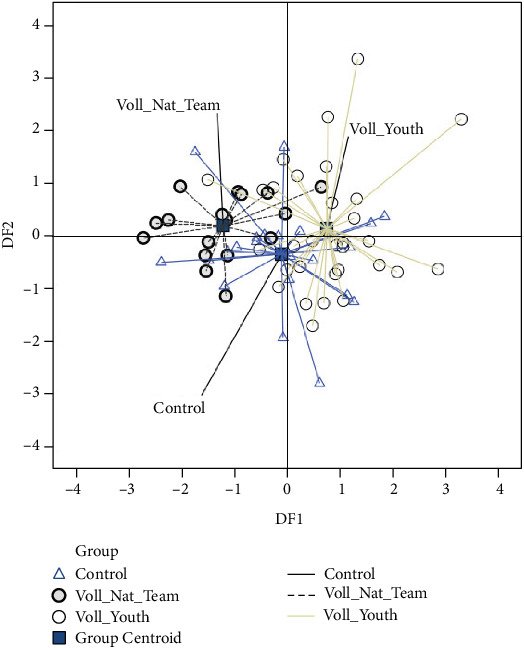
The graphical representation of the centroid position of the examined subsamples.

Figure 3 shows the graphical representation of the centroid position of the examined subsamples in relation to the relevant functions based on the kinematic variables of rapid hand movement.

Based on the results of the descriptive statistical analysis, it was determined that the obtained results of the examined kinematic variables of rapid hand movement have acceptable variation, given the fact that the coefficient of variation is in the range from 7.87 to 45.00 for *t*_2_ in Voll_Youth and GA_2_ in control samples, respectively. The results of the Kolmogorov-Smirnov goodness-of-fit test indicate that the examined variables are normally distributed on a general level (Table 1). The results of Box's test of equality of covariance matrices have shown that the multiple distribution of the examined groups is similar on a statistically significant level (*M* = 78.488, *F* = 1.605, *p* = 0.008). On the basis of the aforementioned, it can be argued that the obtained results have average homogeneity [29] and normal distribution and belong to the same measurement area which makes them representative in terms of further scientific interpretation.

The discriminant analysis has identified two functions, DF_1_ and DF_2_, that explain 91.9 and 8.1% of the variance, respectively. It was determined that DF_1_ is statistically significant (*p* ≤ 0.000). This function is composed of the variables *t*_1_ and *t*_2_. The second function DF_2_ is composed of the variables *A*_1_, *A*_2_, GA_1_, and GA_2_. DF_2_ reached a *p* value of 0.616, thus yielding nonsignificant results (Table 2). This indicates that the differences between the examined subsamples originate from the variables grouped in DF_1_, i.e., the first function. The centroid positions of the examined groups control, Voll_Nat_Team, and Voll_Youth in relation to the function DF_1_ are -0.112, -1.220, and 0.748, respectively (Figure 3). These results show that, in relation to DF_1_, the Voll_Nat_Team group centroid position is shifted with -1.968 and -1.108 standard deviation values from the Voll_Youth and the control group, respectively. The difference between control and Voll_Youth is -0.860. The second discriminant function (DF_2_) did not show a significant difference between the observed groups; thus, the centroid positions of the groups in relation to this function are similar (Figure 3). The factors with the greatest discriminative value among the groups represent the temporal characteristics of the rapid hand movement, i.e., the time elapsed between the onset of the movement and the first (*t*_1_) and second (*t*_2_) tap, as shown in Table 3.

Regarding the efficiency of the IMU-based measurement system in relation to the discrimination of the examined subsamples from the aspect of kinematic characteristics relevant for the rapid hand movement, it was determined that it was 65.7% overall (Table 4). It should be pointed out that the highest accuracy of classification (80.6%) was determined in the subsample of young volleyball players (Voll_Youth), while players in the control group were classified as having the lowest accuracy (40.9%). Based on the kinematic characteristics of rapid hand movement, 36.4 and 22.7% of the control group was classified in the subsamples Voll_Youth and Voll_Nat_Team, respectively (Table 4). For the subsample Voll_Nat_Team, the discriminative efficiency was 70.6%, or 88.2% when taking into account the participants classified in the Voll_Youth group.

The presented results show the potential of IMU sensors in relation to the measurement of rapid movement kinematics. The discriminative nature of the obtained results indicates the applicability of such systems for the purposes of assessment, monitoring, and even selection of athletes.

## 4. Conclusions

The aim of this paper was to determine the discriminative potential of IMU sensor technology in detecting single rapid movement variables/characteristics in females with different training backgrounds and specialization. Rapid hand movement properties, i.e., relevant kinematic variables in relation to different groups of participants, were examined. The measurement of the kinematic variables was performed using a standard hand tapping test. The sample in this research included a total of 70 female participants and was divided into 3 subsamples, of which one included physically active controls, while the other two consisted of the members of the Republic of Serbia national volleyball team and national-level young volleyball players, respectively. The discriminant analysis was used in order to define the centroid location, as a group standardized multivariate score, as well as the structure of the extracted functions and group differences between the respective subsamples. The discriminant analysis has identified two functions, DF_1_ and DF_2_, that explain 91.9 and 8.1% of the variance, respectively. The differences between the examined subsamples originate from the variables grouped in extracted function DF_1_, which was statistically significant at the level *p* ≤ 0.000. In relation to this function, the Voll_Nat_Team group centroid position was shifted with -1.108 standard deviation values from the control and -1.968 standard deviation values from the Voll_Youth group. The difference between the control and Voll_Youth groups was -0.860 standard deviation value. The factors with the greatest discriminative potential among the groups are the variables of the temporal characteristics of the rapid hand movement, i.e., the time elapsed between the onset of the movement and the first and second tap, as defined by the variables *t*_1_ and *t*_2_. The established findings clearly indicate that IMU sensors are practically applicable in this context and can be included as a new technology used for the purposes of assessment, monitoring, and selection of athletes.

## Figures and Tables

**Table 1:** Basic descriptive statistics of the examined variables in relation to the research subsamples with the results of the one-sample Kolmogorov-Smirnov test.

Control									
	*N*	Mean	SEM	StDev	cV%	Min	Max	K-S Z	Sig.
*t*_1_ (s)	22	0.23	0.01	0.03	14.20	0.19	0.29	0.611	0.849
*t*_2_ (s)	22	0.43	0.01	0.05	12.50	0.34	0.54	0.741	0.642
*A*_1_ (g_0_)	22	3.87	0.25	1.17	30.23	2.02	6.23	0.351	1.000
*A*_2_ (g_0_)	22	8.33	0.44	2.06	24.75	5.34	12.24	0.713	0.689
GA_1_ (g_0_·s^−1^)	22	70.94	5.21	24.42	34.42	36.00	122.13	0.834	0.491
GA_2_ (g_0_·s^−1^)	22	211.73	20.31	95.27	45.00	84.34	485.88	0.961	0.314
Voll_Nat_Team									
	*N*	Mean	SEM	StDev	cV%	Min	Max	K-S Z	Sig.
*t*_1_ (s)	17	0.21	0.01	0.03	13.92	0.17	0.26	0.590	0.877
*t*_2_ (s)	17	0.40	0.01	0.04	9.63	0.37	0.50	1.190	0.117
*A*_1_ (g_0_)	17	3.88	0.21	0.88	22.63	2.17	5.32	0.563	0.909
*A*_2_ (g_0_)	17	8.35	0.46	1.91	22.88	4.39	12.07	0.440	0.990
GA_1_ (g_0_·s^−1^)	17	57.30	5.81	23.97	41.84	23.59	109.81	0.433	0.992
GA_2_ (g_0_·s^−1^)	17	229.26	17.62	72.63	31.68	142.95	394.64	0.775	0.586
Voll_Youth									
	*N*	Mean	SEM	StDev	cV%	Min	Max	K-S Z	Sig.
*t*_1_ (s)	31	0.24	0.00	0.03	11.52	0.18	0.30	0.679	0.746
*t*_2_ (s)	31	0.45	0.01	0.04	7.87	0.40	0.52	0.815	0.520
*A*_1_ (g_0_)	31	3.78	0.18	0.99	26.25	2.48	5.89	0.684	0.737
*A*_2_ (g_0_)	31	8.94	0.43	2.42	27.04	4.90	14.16	0.725	0.669
GA_1_ (g_0_·s^−1^)	31	72.34	4.63	25.79	35.65	37.88	154.98	0.908	0.382
GA_2_ (g_0_·s^−1^)	31	252.19	17.87	99.51	39.46	96.47	520.85	0.754	0.620

**Table 2:** The summary of canonical discriminant functions and general intergroup differences.

Eigenvalues				
Function	Eigenvalue	% of variance	Cumulative %	Canonical correlation
1	0.641	91.9	91.9	0.625
2	0.057	8.1	100	0.231
Wilks' lambda				
Test of function(s)	Wilks' lambda	Chi-square	df	Sig.
1	0.577	35.492	12	0.000
2	0.946	3.550	5	0.616

**Table 3:** The structure matrix.

	Function	
	DF_1_	DF_2_
*t*_1_	0.516	-0.007
*t*_2_	0.408	-0.209
*A*_1_	0.145	0.654
*A*_2_	0.295	-0.412
GA_1_	0.144	0.318
GA_2_	-0.056	-0.093

**Table 4:** Classification results.

		Groups	Predicted group membership			Total
			Control	Voll_Nat_Team	Voll_Youth	
Original	Count	Control	9	5	8	22
		Voll_Nat_Team	2	12	3	17
		Voll_Youth	5	1	25	31
	%	Control	40.9	22.7	36.4	100
		Voll_Nat_Team	11.8	70.6	17.6	100
		Voll_Youth	16.1	3.2	80.6	100

65.7% of the original grouped cases were correctly classified.

## Data Availability

The data used to support the findings of this study are available from the corresponding author upon request.
